# Pharmacokinetics and bioequivalence evaluation of two oral formulations of cotrimoxazole tablets in healthy Chinese volunteers under fasting conditions

**DOI:** 10.1186/s40360-024-00743-9

**Published:** 2024-02-27

**Authors:** Xu Zuo, Xin Zhao, Jinjin Shi, Tiandong Zhang

**Affiliations:** Phase I clinical trial research laboratory, Xinxiang central hospital, The Fourth Clinical College of Xinxiang Medical University, Xinxiang, China

**Keywords:** Bioequivalence, Cotrimoxazole, Pharmacokinetics, Safety

## Abstract

This bioequivalence study was conducted to evaluate two oral formulations of cotrimoxazole tablets in healthy Chinese subjects. All 26 subjects recruited to this study were randomly and evenly classified into two groups and received a single dose (sulfamethoxazole: 400 mg and trimethoprim: 80 mg) of test cotrimoxazole tablets (generic drug) or reference cotrimoxazole tablets (branded drug). After a 7-day washout period, these subjects received one dose of reference drug or test drug. Blood samples were collected from participants before and up to 48 h after dosing to assess the concentration of sulfamethoxazole (SMX) and trimethoprim (TMP) in plasma and a plasma concentration-time curve was drawn. Then, the pharmacokinetics parameters were calculated accordingly. Our data revealed that there were no significant differences observed in the maximum plasma concentration (Cmax), area under the curve from time 0 to the last measurable concentration (AUC0-t), and area under the curve from time 0 to infinity (AUC0-∞) between the two formulations. For SMX, the 90% confidence intervals (CI) of the geometric mean ratio for Cmax, AUC0-t, and AUC0-∞ were 104.03-113.92%, 100.46-103.70%, and 100.41-103.81%, respectively. Similarly, for Trimethoprim (TMP), the 90% CI ranged from 98.54 to 106.95% for Cmax, from 99.31 to 107.68% for AUC0-t, and from 99.49 to 107.55% for AUC0-∞. Importantly, all these 90% CI values fell within the range of 80.00–125.00%, indicating that the test drug is bioequivalent to the reference drug. Furthermore, throughout the entire trial, no suspected serious adverse events were reported, indicating the safety profile of the newly developed generic cotrimoxazole. In summary, our study demonstrates that the newly developed generic formulation of cotrimoxazole is bioequivalent to the branded formulation under fasting conditions.

## Introduction

Bacteria are ubiquitous microorganisms found throughout the Earth and constitute a significant portion of the prokaryotic microbial population. They can have both beneficial and detrimental effects on animals. In the case of gut bacteria, they are considered essential for maintaining health by participating in metabolic processes, promoting the production of anti-inflammatory cytokines, and regulating the immune system [1]. However, certain pathogenic bacterial species can cause severe symptoms and illnesses in humans when infection occurs [2]. For example, infection of Streptococcus may cause pneumonia, sepsis, and so on. Bacterial infections occur when these organisms enter the body through wounds in the skin, airways, and other routes. It is crucial to promptly seek appropriate treatment for bacterial infections as untreated infections pose a serious threat to health.

Antibiotics are commonly employed to combat bacterial infections by directly eliminating or inhibiting the growth of bacteria, providing the immune system with time to fight against the infection [3]. Two types of antibiotics, namely Sulfamethoxazole (SMX) and Trimethoprim (TMP), are frequently used to treat various bacterial infections. These include Staphylococcus aureus, Streptococcus pyogenes, Streptococcus pneumoniae, and others [4]. SMX and TMP exert their antibacterial effects by targeting different steps in the folate synthesis pathway [5]. SMX inhibits the activity of dihydropteroate synthetase, an enzyme responsible for converting para-aminobenzoic acid and dihydropteroate diphosphate into dihydrofolic acid or dihydrofolate [5]. On the other hand, TMP acts by inhibiting the activity of dihydrofolate reductase, thus reducing the production of tetrahydrofolic acid [5]. The cotrimoxazole tablet, available in strengths of 400 mg/80 mg and 800 mg/160 mg, combines SMX and TMP in a ratio of 5:1. This formulation was initially developed by Sun Pharmaceutical Industries Inc. and received clinical approval in 1973 [6]. It is widely utilized in clinical practice for infection prevention and treatment [2].

A generic cotrimoxazole tablet (400 mg/80 mg) was recently developed by TEYI Pharmaceutical group Co.,LTD (Guangdong, China). In adherence to the “Opinions of The General Office of the State Council on the Consistency Evaluation of the Quality and Efficacy of Generic Drugs” (No. 106, 2016) set by the National Medical Products Administration (NMPA), it is necessary to conduct a bioequivalence (BE) study to ensure the consistency of our generic drug with the brand drug. According to the Catalog of Generic Reference Preparations (Batch 7, https://www.nmpa.gov.cn/directory/web/nmpa/zhuanti/ypqxgg/ggzhcfg/20170721091801488.html, Last access data 3 Mar, 2023), cotrimoxazole tablet (trade name: BACTRIM®, 400 mg/80 mg) manufactured by Sun Pharmaceutical Industries Inc. were selected as reference product. In this study, blood samples will be collected from healthy Chinese participants after the administration of both the generic and branded cotrimoxazole tablets under fasting conditions. These blood samples will be analyzed using a validated liquid chromatography tandem mass spectrometry (LC-MS/MS) method to measure the concentrations of SMX and TMP in the blood. Subsequently, the relevant pharmacokinetic (PK) parameters will be calculated to evaluate the bioequivalence of the generic drug compared to the branded drug.

## Subjects and methods

### Study drugs

The Cotrimoxazole generic (or test, T) product (400 mg/80 mg, batch number 11,180,703, content of SMX is 99.80%, content of TMP is 100.8%, expiry date June, 2020) was manufactured by TEYI Pharmaceutical group Co., LTD. The Cotrimoxazole branded (or reference, R) product (400 mg/80 mg, batch number 6,842,001, content of SMX is 99.07%, content of TMP is 99.10%, expiry date February, 2020) purchased from Sun Pharmaceutical Industries Inc. was used as reference drug according to the guideline released by China’s NMPA [7]. Drugs were stored avoid of light and in sealed (temperature 20–25 ℃).

### Study design

This study was conducted at the phase I clinical trial research laboratory of Xinxiang Central Hospital. The study protocol received approval from the Ethics Committee of Xinxiang Central Hospital (2018-044). Registration for this study was completed on the “Chemical drug bioequivalence trial record information platform” (www.chinadrugtrials.org.cn, 29/12/2018, CTR20182518). Throughout the trial, strict adherence was maintained to the Helsinki Declaration and relevant national or domestic laws and regulations. The sample size calculation was performed using the PASS software (version 11.0.7) considering the following criteria: (1) an intra-coefficient of variation of the maximum serum drug concentration (Cmax) of 23% at a significance level of 0.05 and a confidence level of 80%, (2) a geometric mean ratio of the test drug and reference drug within the range of 0.95–1.05, and (3) the bioequivalence range for pharmacokinetic parameters set at 80-125%. Based on these considerations, the estimated sample size was determined to be 24 using the formula described by Pourhoseingholi et al. [8]. To account for potential dropouts, the final sample size for this study was set at 26.

The study design followed a single-center, randomized, open-label, two-drug, two-period, crossover, single-dose bioequivalence study conducted under fasting conditions, in accordance with the relevant guidelines [7, 9, 10]. The “Technical guidelines for research on bioavailability and bioequivalence of chemical drug preparations in humans” [11] and “Technical guidelines for human bioequivalence study of chemical generic drugs with pharmacokinetic parameters as the end point evaluation index” [12] issued by NMPA were considered. The washout period between the two periods of the study was set to be at least 7 times the terminal elimination half-life. The half-life of SMX is approximately 10 h, while for TMP, it ranges from 8 to 10 h [13]. Therefore, a washout period of 7 days was chosen for this study.

### Subjects

A total of 58 Chinese individuals were screened for eligibility, out of which 26 subjects were selected for participation in this study. The age range of the recruited subjects was between 18 and 65 years, inclusive of the boundary values. Their body mass index (BMI) fell within the range of 19 to 26 kg/m^2^. Female participants were required to have a body weight equal to or above 45 kg, while male participants needed to have a body weight of 50 kg or higher.

Prior to enrollment, the investigators collected and examined the subjects’ medical examination results, medical history, and clinical laboratory test results. It was ensured that these individuals did not have any abnormalities of clinical significance. Written informed consent was obtained from all subjects, and they had a comprehensive understanding of the study procedures before the commencement of the experiment.

Subjects with deficiencies in Glucose-6-phosphate dehydrogenase or folate, as well as those exhibiting symptoms of porphyria, were excluded from participation in this study. Additionally, individuals meeting the following criteria were also excluded: (1) significant abnormalities in their laboratory test results; (2) a history of mental illness, drug abuse, or consumption of more than 14 units of alcohol per week within the three months prior to screening; (3) a known allergy to SMX, TMP, furosemide, sulfone, thiazide diuretics, sulfonylurea, or carbonic anhydrase inhibitors; (4) intolerance to intravenous indwelling needles or blood phobia; (5) consumption of more than 8 cups (250 ml per cup) of tea, coffee, or caffeinated beverages per day within the three months prior to screening; (6) dietary requirements that prevented compliance with the provided regulations; (7) any other cases deemed inappropriate by the principal investigators.

### Study procedure

The enrolled subjects were admitted to our center one day prior to drug administration and underwent a fasting period of 10 h. On day 1, the subjects were randomly divided into two groups: the T-R group and the R-T group. Each individual in both groups orally received the assigned drug (either T or R) with 240 ml of water in the morning. Four hours and ten hours after drug administration, the subjects were provided with lunch and dinner, respectively. During the hour before and after drug administration, except for taking the drug with water, subjects were not permitted to consume any other liquids. Additionally, they were advised to refrain from engaging in extensive physical activities for four hours after taking the drug. On day 7, all subjects returned to the trial center for further assessments, including inquiries and vital sign examinations. After fasting for 10 h, the subjects followed the scheduled protocol and took another pill as instructed.

### Blood sampling

After oral administration, both SMX and TMP are efficiently absorbed, making it possible to evaluate their bioequivalence by measuring blood drug concentrations. Venous blood samples of 4 ml were collected from the upper extremities before and after dosing. To ensure that the sampling time points cover the drug’s absorption, distribution, and elimination phases, the final time point should be no less than 3 times the drug’s half-life. Therefore, blood samples were collected at the following time points: 0 h (within 60 min before dosing), 10 min, 20 min, 30 min, 45 min, 1 h, 1.25 h, 1.5 h, 2 h, 2.5 h, 3 h, 3.5 h, 4 h, 5 h, 6 h, 8 h, 12 h, 24 h, 36 h, and 48 h after dosing. To obtain plasma samples, all blood samples were centrifuged at 1,700 g for 10 min at 4 °C within 1 h of collection. The plasma samples were then stored at -60 °C for subsequent analyses. The concentrations of SMX and TMP in the plasma were determined using a validated LC-MS/MS method using Shimadzu UPLC-30 AD Ultra High-Performance Liquid Chromatography System equipped with Agilent EC-C18 50*4.6 mm 2.7 μm column and Applied Biosystems Triple Quad-4500 Triple Quadrupole Mass Spectrometer performed by a contracted company. The peak information used for sample identification was as follows: m/z 254.1—155.9 for SMX, m/z 291.4—123.3 for TMP, m/z 257.8—159.9 for SMX-d4, m/z 194.2—230.1 for TMP-d3. The linear concentration range for SMX was 200 − 40,000 ng/ml, while for TMP, it was 8 − 1,600 ng/ml.

### Pharmacokinetic parameters

The pharmacokinetic (PK) analysis was performed using the noncompartmental model in Phoenix WinNonlin version 8.0 (Certara, Princeton, New Jersey). Various parameters were calculated to assess the PK characteristics of the drugs under investigation. These parameters included C_max_ (maximum concentration), T_max_ (time to reach C_max_), AUC_0 − t_ (area under the concentration-time curve from dosing to a specific time point, t), AUC_0−∞_ (area under the curve extrapolated to infinite time), AUC__%Extrap_ (percentage of AUC extrapolated), T_1/2_ (terminal elimination half-life), and λz (terminal elimination rate constant). C_max_ and T_max_ were directly obtained from the concentration-time curve, while the remaining parameters were calculated using previously described methods [14, 15]. The geometric mean ratio of C_max_, AUC_0 − t_, and AUC_0−∞_ between the test and reference drugs was determined, along with the corresponding 90% confidence interval (CI). If the 90% CI fell within the range of 80-125%, it was considered indicative of bioequivalence between the test drug and the reference drug.

### Safety assessments

To assess the safety of the drugs, various measures were taken, including body vital examination, physical examinations, 12-lead electrocardiogram (ECG), and laboratory tests such as blood routine examination, urine routine examination, and blood biochemical tests. These assessments were conducted to monitor any potential adverse events (AEs) that may arise during the study. During the evaluation of AEs, their severity was determined in accordance with the Common Terminology Criteria for Adverse Events 5.0 [16].

### Statistical analyses

Data were presented as mean ± standard deviation (SD). Statistical analyses were conducted at SAS (version 9.4, SAS Institute, Cary, North Carolina) software. Analysis of variance (ANOVA) was used to analyze the difference of main PK parameters (C_max_, AUC_0 − t_, and AUC_0−∞_) after logarithmic transformation. Mean difference in T_max_ values were compared using a nonparametric Wilcoxon signed rank sum test. P value less than 0.05 was to indicate statistically significant.

## Results

### Study subjects’ demographics

**Fig. 1:**
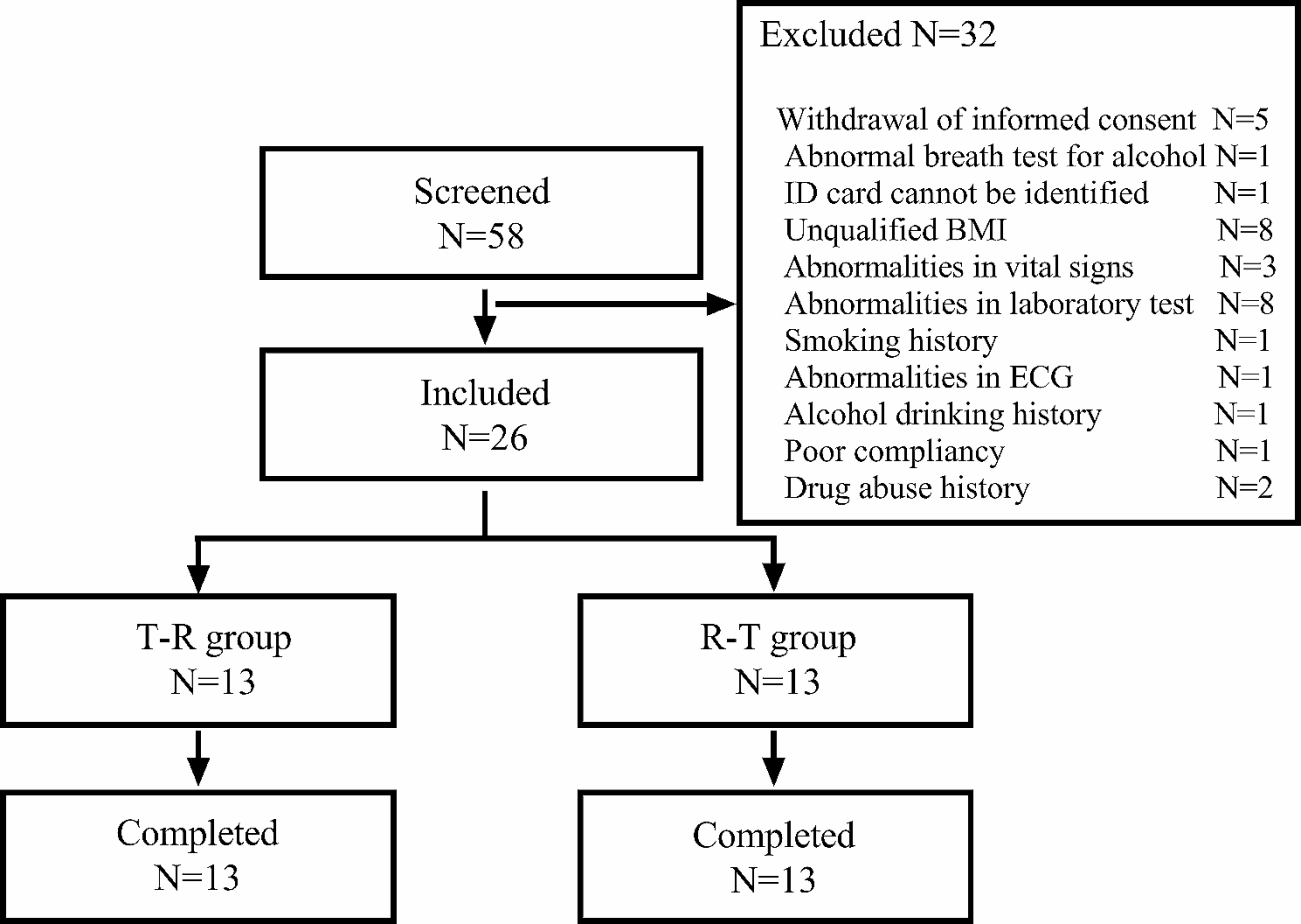
Screening procedure of the healthy subjects in this study

A total of 58 subjects were initially screened based on the predetermined criteria. After careful evaluation, 26 subjects were enrolled in the study following sample size estimation and calculation. Thirty-two subjects were excluded from participation as they did not meet the enrollment criteria. The screening process and the reasons for subject dropout are presented in Fig. 1. Among the 26 enrolled subjects, 18 were male and 8 were female. Demographic data for these subjects were collected and summarized in Table 1.

**Table 1:** Demographic Characteristics of All Subjects

Demographic	Fasting (*N* = 26)	Range
Age, y, mean (SD)	32.04(10.86)	19–55
Sex, N (%)		
Male	18(69.2)	
Female	8(30.8)	
Body weight, kg, mean (SD)	64.63(8.70)	49.20–82.70
Height, cm, mean (SD)	168.94(8.67)	153.50-183.50
BMI, kg/m^2^, mean (SD)	22.59(2.08)	19.00-25.80
Race, N (%)		
Han	26(100)	

BMI, body mass index; SD, standard deviation

### PK assessments

**Fig. 2:**
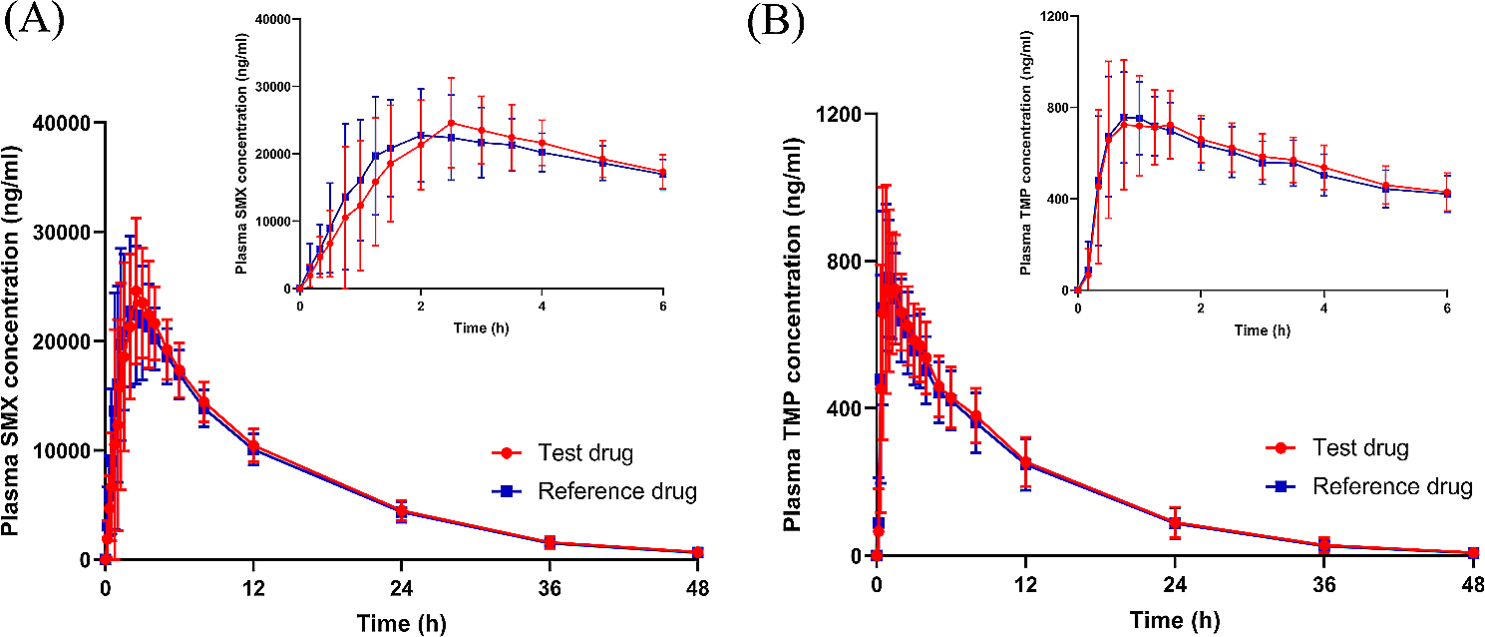
Mean (± standard error) plasma concentration-time curves for (**A**) SMX and (**B**) TMP in fasting study of test drug or reference drug. The error bars are standard deviation. SMX: sulfamethoxazole; TMP: Trimethoprim

All 26 subjects successfully completed the entire trial process, allowing for the inclusion of their data in the PK concentration set (PKCS), PK parameter set (PKPS), and bioequivalence set (BES) analyses. The mean plasma concentration-time curves for both SMX and TMP were depicted in Fig. 2a and b, showcasing the overall drug profiles. Table 2 summarized the PK parameters for subjects receiving either the test or reference drug administration. It is important to note that data from one subject was excluded from the analysis due to the consumption of water within 1 h after drug administration, which could potentially affect the drug’s absorption. The calculated ratios of AUC_0 − t_ and AUC_0−∞_ for both SMX and TMP, following administration of the test or reference drug, were found to be above 80%. This indicates that the 48-hour time point for blood sample collection was appropriate. Additionally, the T_1/2z_ for SMX was approximately 8 h, while for TMP, it was around 7 h (as shown in Table 2). Considering that the 48-hour sample collection falls beyond 5 half-life times for both SMX and TMP, it further confirms the suitability of our chosen sampling time points. Moreover, these findings validate the appropriateness of the 7-day wash-out period, as it exceeds the 7-fold duration of the drug’s half-life. This ensures an adequate elimination of the previously administered drug before the subsequent dosing, minimizing any potential carryover effects.

**Table 2:** Primary PK evaluation (PK analysis set)

Parameter (units)	Sulfamethoxazole		Trimethoprim	
	mean ± SD (% CV)		mean ± SD (% CV)	
	Test (*N* = 25)	Reference (*N* = 25)	Test (*N* = 26)	Reference (*N* = 25)
T_max_ (h)*	2.5 (0.75,4.00)	1.5 (0.75,5.00)	0.75 (0.33,2.00)	1.00 (0.33,3.50)
C_max_ (ng/mL)	29,500 ± 5800 (19.7)	27,100 ± 5620 (20.7)	894 ± 208(23.2)	863 ± 163 (18.9)
AUC_0 − t_ (ng·h/mL)	330511.74 ± 44951.68 (13.6)	323200.10 ± 38368.41 (11.87)	8362.34 ± 1999.94 (23.92)	8120.30 ± 2042.16 (25.15)
AUC_0−∞_ (ng·h/mL)	339444.27 ± 47932.52 (14.12)	331787.30 ± 40534.69 (12.22)	8536.13 ± 2059.01 (24.12)	8286.28 ± 2095.23 (25.29)
λz (1/h)	0.08 ± 0.01 (13.11)	0.08 ± 0.01 (13.35)	0.10 ± 0.01 (14.67)	0.10 ± 0.02 (15.76)
T_1/2z_ (h)	8.74 ± 1.15 (13.11)	8.80 ± 1.12 (12.76)	7.17 ± 1.11 (15.49)	7.18 ± 1.18 (16.47)

Tmax, time to reach maximum concentration, shown as median (min, max); Cmax, maximum plasma drug concentration; AUC_0 − t_, area under the plasma concentration-time curve from time 0 to the last measurable concentration; AUC_0−∞_, area under the plasma concentration-time curve from time 0 to infinity; PK, pharmacokinetic; T_1/2z_, elimination half life

* Median (Min, Max)

### Bioequivalence analysis

Based on the acquired PK parameters, we proceeded with statistical analyses to evaluate the bioequivalence of the test and reference drugs, as presented in Table 3. For SMX, the geometric mean ratios of C_max_, AUC_0 − t_, and AUC_0−∞_ were calculated to be 108.86%, 102.07%, and 102.09%, respectively. The corresponding 90% confidence intervals (CI) were determined as 104.03-113.92%, 100.46-103.70%, and 100.41-103.81%. Similarly, for TMP, the geometric mean ratios of C_max_, AUC_0 − t_, and AUC_0−∞_ were found to be 102.66%, 103.41%, and 103.44%, respectively, with 90% CIs of 98.54-106.95%, 99.31-107.68%, and 99.49-107.55%. Importantly, all these calculated ratios and their corresponding 90% CIs fell within the predefined bioequivalence range of 80.00-125.00%. This indicates that there were no significant differences observed between the test and reference drugs in terms of C_max_, AUC_0 − t_, and AUC_0−∞_ for both SMX and TMP. However, a significant difference was observed in T_max_ between the test and reference drugs for SMX, with the test drug exhibiting a delay of 1 h compared to the reference drug. This difference was statistically significant as determined by the non-parametric Wilcoxon signed rank sum test (Table 4). In contrast, no significant difference was found in T_max_ for TMP (Table 4). Collectively, these findings suggest that under fasting conditions, the test drug is bioequivalent to the reference drug, as evidenced by the lack of significant differences in C C_max_, AUC_0 − t_, and AUC_0−∞_ for both SMX and TMP. The observed delay in Tmax for SMX between the test and reference drugs warrants further investigation but does not undermine the overall conclusion of bioequivalence.

**Table 3:** Bioequivalence analysis Under the Fasting Study

Group	Parameter (units)	GM		GMR	CV%	90% CI
		Test	Reference	(Test/Reference) %		
Sulfamethoxazole *N* = 26	C_max_ (ng/mL)	28997.53	26636.93	108.86	9.6	104.03%~113.92%
	AUC_0 − t_ (ng·h/mL)	327608.67	320972.67	102.07	3.34	100.46%~103.70%
	AUC_0−∞_ (ng·h/mL)	336265.40	329377.49	102.09	3.51	100.41%~103.81%
Trimethoprim *N* = 26	C_max_ (ng/mL)	870.61	848.09	102.66	8.65	98.54%~106.95%
	AUC_0 − t_ (ng·h/mL)	8141.70	7873.25	103.41	8.53	99.31%~107.68%
	AUC_0−∞_ (ng·h/mL)	8309.77	8033.24	103.44	8.21	99.49%~107.55%

C_max_, maximum plasma drug concentration; AUC_0 − t_, area under the plasma concentration-time curve from time 0 to the last measurable concentration; AUC_0−∞_, area under the plasma concentration-time curve from time 0 to infinity; CV%, intrasubject coefficient of variation; GM, geometric mean; GMR, geometric mean ratio; N(T), number of healthy subjects in test drug treated group; N(R), number of healthy subjects in reference drug treated group

**Table 4:** Nonparametric test of T_max_ of Sulfamethoxazole and Trimethoprim

	Sulfamethoxazole					Trimethoprim				
	N	Mean	SD	Median	P value	N	Mean	SD	Median	P value
Reference drug	26	1.83	1.01	1.50		26	1.00	0.65	1.00	
Test drug	26	2.14	0.75	2.50		26	0.96	0.53	0.75	
Reference drug-Test drug	26	0.32	0.79	-0.50	0.0056	26	0.04	0.60	0.00	0.8573

SD: standard deviation

### Safety analysis

Throughout the entire trial process, no suspected serious adverse events were reported, and no participants dropped out due to adverse events. Out of the 26 subjects enrolled, a total of 8 adverse events occurred in 6 individuals, resulting in an incidence rate of 23.1% (6/26). Importantly, all these adverse events were classified as mild in severity, and no specific treatment measures were required for their management. Among the reported adverse events, 3 were deemed “possibly related” to the drug, and all of them occurred when the reference drug was administered to a single subject. The details of these adverse events can be found in Table 5. It is worth noting that one subject experienced fainting prior to drug dosing during the first period and was therefore not included in either the test drug or reference drug group. When considering the administration of the test drug, 2 adverse events occurred (2/26, 7.7%), while 5 adverse events occurred during the administration of the reference drug (5/26, 11.5%). Overall, these data indicate that the oral administration of a single-dose Cotrimoxazole tablet (400 mg/80 mg) was found to be safe and well-tolerated in both male and female healthy Chinese subjects under fasting conditions. The low incidence of adverse events and the mild severity of those reported further support the safety profile of the drug.

**Table 5:** Summary of TEAEs (safety analysis set)

Most common TEAEs	Test (*N* = 26)		Reference(*N* = 26)	
	Case(N)	Number of cases (%)	Case(N)	Number of cases (%)
Infectious and invasive diseases, N (%)	1	1(3.8)	0	0(0.0)
Fever Blisters	1	1(3.8)	0	0(0.0)
Laboratory findings	0	0(0.0)	1	1(3.8)
Hematemesis	0	0(0.0)	1	1(3.8)
Nervous system disorders	1	1(3.8)	0	0(0.0)
Dizziness	1	1(3.8)	0	0(0.0)
Diseases of the respiratory system, chest, and mediastinum	0	0(0.0)	1	1(3.8)
Hiccup	0	0(0.0)	1	1(3.8)
Kidney and urinary diseases	0	0(0.0)	1	1(3.8)
Oliguria	0	0(0.0)	1	1(3.8)
Gastrointestinal diseases	0	0(0.0)	1	1(3.8)
Abdominal Discomfort	0	0(0.0)	1	1(3.8)
Eye organ diseases	0	0(0.0)	1	1(3.8)
Asthenopia	0	0(0.0)	1	1(3.8)

TEAEs, treatment-emergent adverse event

## Discussion

Folate is an essential metabolite required for DNA and RNA synthesis, playing a critical role in cell growth [17]. Consequently, agents targeting the folate synthesis pathway, known as antifolates, have been developed and extensively used in the fields of bacterial infection and cancer treatment [18–20]. Methotrexate, for instance, inhibits the production of tetrahydrofolate by directly targeting and inhibiting the activity of folate reductase. As a result, it is widely employed in the treatment of autoimmune diseases and cancer. Furthermore, antifolates have found widespread application in patient treatment for bacterial infections. Sulfamethoxazole (SMX) and trimethoprim (TMP) are two commonly used antibiotics for the treatment of various bacterial infections. These agents act by inhibiting two consecutive steps in the folate synthesis pathway, potentially leading to improved treatment outcomes. The co-administration of SMX and TMP is believed to slow down the development of drug resistance in comparison to using either SMX or TMP alone. Based on these facts, the Cotrimoxazole Tablets have been developed and yet achieved great success in clinical.

To address the need for accessible and cost-effective antibacterial drugs due to the prevalence of bacterial infections, we conducted a clinical trial to evaluate the bioequivalence of a generic Cotrimoxazole Tablets with the branded drug. The aim was to support the clinical use of the generic drug. Our data showed that there is no significant difference in the C_max_ of both SMX (29,500 ng/ml for test drug and 27,100 ng/ml for reference drug) and TMP (894 ng/ml for test drug and 863 ng/ml for reference drug) of test drug and reference drug. With the concentration-time curve, we calculated AUC_0 − t_ and AUC_0−∞_ for SMX and TMP. Similar to the C_max_ data, no significant differences were observed in these parameters between the test and reference drugs. These findings suggest that the absorption of both drugs in healthy subjects is comparable. However, it is worthwhile to mention that T_max_ for SMX in the test drug was higher than reference drug (2.5 h vs. 1.5 h), and this difference was statistically significant. Nevertheless, the observed T_max_ for the test drug still falls within the range of 1–4 h as documented in the Cotrimoxazole Tablets leaflets [21]. In the case of TMP, no significant difference in T_max_ was observed between the test and reference drugs. Importantly, we calculated the 90% CI for the key PK parameters (C_max_, AUC_0 − t,_ and AUC_0−∞_) in accordance with relevant guidelines. Our data clearly demonstrated that all these 90% CI data fell in the 80.00-125.00% range, which support the idea that our generic Cotrimoxazole Tablets developed by TEYI Pharmaceutical group Co., LTD. are bioequivalent to the brand drug developed by Sun Pharmaceutical Industries Inc.Another crucial aspect to consider before a drug can be used in humans is its safety profile. Therefore, we carefully collected and evaluated adverse events that occurred during the trial following the administration of both the test and reference drugs. A total of 8 adverse events were reported, and our research group diligently assessed and graded each event according to relevant guidelines. Fortunately, all these adverse events were determined to be mild in nature. Furthermore, out of the 8 events, only 3 occurred in a single subject and were deemed possibly related to the drug administration. Importantly, these events were exclusively observed during the administration of the reference drug. Overall, our data clearly demonstrate that the generic drug is safe for the health of Chinese subjects participating in the study.

Finally, we also compared our research data with the previous studies conducted at other country and region [22, 23] and presented these data in Table 6. The T_max_ and T_1/2_ of SMX is similar with the previous published data. However, for TMP, our study observed a slightly lower T_max_ compared to the previous studies. The C_max_ of SMX and TMP obtained from our study is also similar with the previous studies after adjusting for dosage variations. For AUC_0 − t,_ we found similarity in the case of SMX compared to the literature data. However, for TMP, our study reported slightly lower values for AUC_0 − t_ compared to the literature. These differences in the data may be attributed to variations in subject populations and reagents used between studies. This comparison indicated that data collected from our data is comparable with previous data, which further remind us that difference race did not have too much influence on the absorption of SMX and TMP. It is important to note that the clinical trial was conducted exclusively under fasting conditions, as per the exemption granted by the NMPA [24].

**Table 6:** Comparison of PK parameters in this study with previous published studies

	Source	N	Dosage	Status	C_max_ (ng/mL)	T_max_ (h)	T_1/2_ (h)	AUC_0 − t_ (h•ng/mL)
Sulfamethoxazole	Test drug in this study	26	400 mg/80 mg	Fasting	29,500 ± 5800	2.14 ± 0.75	8.74 ± 1.15	330511.74 ± 44951.68
	Reference drug in this study	26	400 mg/80 mg	Fasting	27,100 ± 5620	1.83 ± 1.01	8.80 ± 1.12	323200.10 ± 38368.41
	Previous study (Baktar 2011)	10	800 mg/160 mg	Fasting	58,000 ± 6500	1.7 ± 0.7	9.4 ± 1.6	675,490 ± 100,730
	Previous study (UK Public Assessment Report 2015)	-	800 mg/160 mg	Fasting	52801.2 ± 8109.56	-	-	775342.6 ± 85041.99
Trimethoprim	Test drug in this study	26	400 mg/80 mg	Fasting	894 ± 208	0.96 ± 0.53	7.17 ± 1.11	8362.34 ± 1999.94
	Reference drug in this study	26	400 mg/80 mg	Fasting	863 ± 163	1.00 ± 0.65	7.18 ± 1.18	8120.30 ± 2042.16
	Previous study (Baktar 2011)	10	800 mg/160 mg	Fasting	2060 ± 340	2.0 ± 1.2	8.2 ± 3.7	21,740 ± 4060
	Previous study (UK Public Assessment Report 2015)	-	800 mg/160 mg	Fasting	1562.2 ± 288.64	-	-	22981.3 ± 5550.49

## Conclusion

Cotrimoxazole Tablets (400 mg/80 mg) tablet of test and reference drug are bioequivalent in healthy subjects and they show similar pharmacokinetic behavior after dosing. Furthermore, the safety evaluation conducted during the trial demonstrates that both the generic and brand drugs are safe for use in healthy subjects. These findings provide a solid foundation for considering the generic drug as a suitable alternative for antibacterial treatment in clinical settings. By establishing the bioequivalence and safety of the generic drug, our study supports its potential use as an effective and safe option for antibacterial therapy.

## Data Availability

Data are availability from corresponding author upon reasonable request but remain subject to all applicable legal requirements to protect the confidentiality of the study participants? personal information.
